# In vivo screening and evaluation of four herbs against MRSA infections

**DOI:** 10.1186/s12906-017-2001-z

**Published:** 2017-11-23

**Authors:** Najma Arshad, Arifa Mehreen, Iram Liaqat, Muhammad Arshad, Humera Afrasiab

**Affiliations:** 1 Department of Zoology, University of the Punjab, Lahore, Punjab Pakistan; 2 Department of Zoology, Government College University, Lahore, Punjab Pakistan; 3 Department of Zoology, University of Education, Lahore, Punjab Pakistan; 4 Department of Botany, University of the Punjab, Lahore, Punjab Pakistan

**Keywords:** *S. aureus*, *T. vulgaris*, *Z. jujuba*, in vivo, *A. officinalis*, *C. latifolia*, Haematology, Bacterial load, Lesion score

## Abstract

**Background:**

Recently, we reported high in vitro antibacterial efficacy of *Althaea officinalis*, *Ziziphus jujuba, Cordia latifolia* and *Thymus vulgaris* out of a total 21 plants against wide range of bacteria including MRSA. This study was therefore, designed to confirm efficacy of these four herbs against MRSA in an animal model.

**Methods:**

A pilot study was conducted to establish the dose of *S. aureus* (KY698020) required to induce clinical infection. Afterword, in main trial, efficacy of aforementioned plant extracts on the course of sore throat was checked by evaluating general health, gross lesion score, bacterial load and hematology in mice.

**Results:**

Pilot study revealed that 40 μl dose of 10^7^ CFU/ml could induce infection which persist upto 08 days post infection. Mice treated with *T. vulgaris* and *Z. jujuba* showed reduction in gross lesion score of both heart and lungs. Treatment with only some plants could significantly decrease bacterial load of throat (*T. vulgaris*) heart, blood and joint (*C. latifolia*, and *T. vulagris*). Hematological indicators confirmed in vivo control of MRSA infection in all treatment groups except *A. officinalis.*

**Conclusion:**

This is first report confirming in vivo anti-MRSA potential of *C. latifolia* and *T. vulgaris* and highlight the need to explore bioactive constituents of these plants. Moreover, previously reported in vitro antibacterial efficiency of *A. officinalis* could not be validated in current study.

## Background

Herbal medicines have gained popularity throughout world as alternative therapeutics for controlling common infections. The emergence of multiple drug resistant strains of bacteria and high cost of synthetic compounds has directed researchers to look for new therapeutic agents including medicinal plants [1, 2]. Many laboratories are currently involved in documenting application of medicinal plants against infectious and noninfectious diseases through in vitro and in vivo systems [3, 4]. Previously we have described in vitro anti-MRSA potential of 29 herbs, of which *Althaea officinalis*, *Ziziphus jujuba*, *Cordia latifolia* and *Thymus vulgaris* were found superior in controlling MRSA [5].

Ethanol and water extracts of *A. officinalis* flower, its flavonoid hypolaetin 8-glucoside have already been reported to have antibacterial, antifungal, anti-inflammatory, analgesic, anti-ulcer and antiviral activity [6–8]. *Z. jujuba* is reported to be rich in flavonoids, saponins, tannins, vitamin A and B, carbohydrates, calcium, phosphate and iron and has been found effective against multiple ailments [9–15]. *C. latifolia* has also been documented to possess antimicrobial, antibiotic-modifying, anti-inflammatory, antinociceptive, antifertility, toxicity, anti-snake bite, hypolipidemic, immunomodulatory, insecticidal and antioxidant [16–21]. Similarly, therapeutic properties of *T. vulgaris* have been reported by various authors [22–29]. However, previous claims on antimicrobial efficacy of these herbs are solely based on in vitro experimentation which sometimes yield misleading results therefore, verification of efficacy in vivo is considered crucial. This part of study was focused on in vivo validation of the anti-MRSA potential of *C. latifolia*, *A. officinalis*, *T. vulgaris* and *Z. jujuba*.

## Methods

### Bacteria and culture conditions

The *S. aureus* strain KY698020 was isolated from sore throat patients and used in this study. It was characterized on the basis of its anti-biogram and molecular properties. The challenge strain contained *clf A, spa, CPs8, tst, sea, sed* genes and was resistant to methicillin, cefixime, oxacillin, gentamycin and penicillin. The pure culture was kept at −80 °C and refreshed on Mannitol salt agar plate (Oxoid) before use.

### Inoculum preparation

The test strain was refreshed on Lauria Britanica broth at 37 °C for 24 h. For inoculum preparation, culture was centrifuged at 3380×g for 15 min and pellet was washed twice with PBS. The optical density was adjusted at 1 ± 0.02 (at 600 nm) analogous to app. 10^11^ CFU/ml and tenfold dilutions of this suspension was used in mice to establish infection model [30]. The corresponding CFU/ml was confirmed by plating 100 μl of serial dilution of the inoculum on Mannitol salt agar plates.

### Plant extract preparation

Plant extracts were prepared in 99.99% methanol (10 g/100 ml). Extraction was accomplished in a sonicator for 2 h at room temperature. The resulting extracts were filtered, concentrated in a rotary evaporator, dried and stored at 4 °C until further use. These extracts were administered to mice (*Mus musculus*) by force feeding at the rate of 200 mg/ml per kg of body weight.

### Ethical statement

All animals were used in study according to the rulings of the Institute of Laboratory Animal Resources, Commission on Life Sciences, National Research Council (UK). The protocol used was reviewed and approved by the Animal Experiment Administration Committee of University of the Punjab, Lahore, Pakistan.

### Animals and housing conditions

A total of 100 *S. aureus* free mice (*Mus musculus*) were used in this study and maintained in animal house, Department of Zoology. The mice were distributed randomly in isolated metallic cages of size (14″ × 10″ × 7″) under controlled conditions at 30 °C with 12-h variation of light and dark period. Water and feed were provided ad libitum. Adults mice (25–30 g weight, both genders) were acclimatized for 1 week prior to the experiment. Three randomly selected mice were sacrificed and screened for the presence of *S. aureus* in throat, lungs, heart, joints and blood by impression technique on selective medium (Mannitol salt agar supplemented with 2.08 μg/ml of vancomycin).

### Experiment 2 (main study)

#### Animals and grouping

In main study, 42 animals were divided in seven groups (I - VII). To check out the specific objectives, group I and II were kept as negative and positive control respectively, group III received treatment with known antibiotic and group IV – VII served as experimental group and received the calculated dose of methanolic extract of selected plant.

#### Infection and treatment

Animals in the group IV- VII were treated with *A. officinalis*, *C. latifolia*, *T. vulgaris* and *Z. jujuba* with dose of 200 mg/ml per kg of body weight respectively. Infection was given to group II - VII with 40 μl of 4 × 10^7^ CFU/ml through intra-tracheal route. Antibiotic and plant extracts were administered by force feeding through feeding needle once daily. Animals were observed for visible signs, symptoms and mortality on daily basis for a period of 7 days.

#### Sampling

On eighth day post infection, all animals were euthanized by intra peritoneal injection of ketamine (200 mg/kg of body weight) and their blood samples were stored at 4 °C in EDTA vials. Throat, lung, heart, blood and joints were separated aseptically for bacteriological load examination and blood samples were assessed for hematological studies.

### Scoring scheme and laboratory procedures

#### Gross pathological lesion score

Tissue lesions on lung and heart were noticed and scored as follows: Heart: 0 = normal; 1 = vascularization (ii) Lung: 0 = normal; 1 = edema, hyperemia and fibrin in air sacs; 2 = small blood patches; 3 = large blood patches.

### Bacteriological examinations in tissues

#### Qualitative examination (re-isolation of test strain from tissue)

The presence of test strain in throat, lung, heart, blood and joint were determined qualitatively by streaking the samples directly on selected media. The plates were incubated at 37 °C for 24 h and checked for the presence of *S. aureus*.

#### Quantitative examination (bacterial load determination)

Weighed portion of throat, lung, heart, joint and blood was homogenized in 1–2 mL of PBS. About 50 μl of serial dilutions of the mixture were spread on Mannitol salt agar plates to check for bacterial quantification. Data was expressed as CFU/g of issue and CFU/ml of blood.

### Hematology and clinical biochemistry of blood samples

Hematological examination of blood including hemoglobin (Hb), red blood cell count (RBC), packed cell volume (PCV), total leucocyte count (TLC) was done through auto-analyzer. Erythrocytes sedimentation rate (ESR) and differential leukocyte count (DLC) were performed manually.

### Data analysis

The data were analyzed using one-way ANOVA followed by Duncan’s multiple range tests. All analyses were performed in SPSS software for window version 16.0 (SPSS). Probability values ≤0.05 were considered significant.

## Results

### Experiment 1 (pilot study)

#### Establishing an infection model

Mice inoculated with 40 μl of 2 × 10^6^ CFU/ml *S. aureus* remained clinically healthy upto 6 days post infection. Bacteria could not be isolated from throat, liver, lungs, heart, blood and joint after 3 days post infection (dpi) onward. No significant variation in body weight could be observed in the treated and controlled group. No gross lesion in lungs and heart could be observed in autopsy and similarly bacteria could not be isolated from any organ. The mice inoculated with 40 μl of 4 × 10^7^ CFU/ml showed signs of pathological condition with watery nose, lethargic condition and high body temperature. On day seven post infection gross lesions were observed in the lungs of all animals and re-isolation of the *S. aureus* was also recorded from the throat, lungs, heart, joint and blood. In addition, only 1 animal died during the experimental time. The loss in body weight of the mice was also noticed at the end of the experiment. When animals were inoculated with a higher dose of 40 μl of 5 × 10^8^ CFU/ml, all animals showed adverse clinical signs characterized by apathy, lethargy, high body temperature, loss of body weight, ruffled coat and around 50% mice were dead by 4th dpi. The surviving mice were weak and sacrificed at 8th dpi. During necropsy, the gross lesions were observed in lungs (edema and hyperemia), and heart (vascularization). Test strain could be re-isolated from all tissues studied (throat, lungs, heart, blood and joint). All three doses effected the blood parameters including ESR, Hb, RBC, platelet, PCV, TLC, neutrophils, lymphocytes, monocytes and eosinophil (Tables 1 and 2).

**Table 1:** Pilot study: Effects of different doses of *S. aureus* (KY698020) on survival and re-isolation frequency in mice

Parameters	Doses (CFU/ml)		
	5 × 10^8^	4 × 10^7^	2 × 10^6^
Mortality	13/25	4/25	0/25
Re-isolation at day 7 post infection			
Throat	12/12	21/21	1/25
Lungs	12/12	21/21	0/25
Heart	12/12	20/21	0/25
Joint	12/12	20/21	0/25
Blood	12/12	20/21	0/25

**Table 2:** Effect of different doses of *S. aureus* on hematological prameters of Mice

Blood parameter	Dose (CFU/ml)		
	5 × 10^8^	4 × 10^7^	2 × 10^6^
ESR (mm/h)	31.00 ± 0.57^a^	27.80 ± 2.35^a^	2.33 ± 0.66^b^
Hb (g/dl)	7.20 ± 0.60^c^	9.56 ± 0.54^b^	15.33 ± 0.33^a^
RBC/mm3	2.26 ± 0.21^a^	4.17 ± 0.66^b^	5.36 ± 0.166b
Platelet*1000	379.67 ± 11.1	492.60 ± 1.35	333.33 ± 16.66
PCV (%)	25.66 ± 2.18^a^	35.46 ± 2.95^b^	42.46 ± 0.73^b^
TLC*1000	9.33 ± 0.66	5.16 ± 2.26	1.97 ± 1.93
Neutrophil (%)	34.33 ± 4.70^a^	26.00 ± 4.58^ab^	11.67 ± 1.66^b^
Lymphocyte (%)	66.66 ± 3.33	70.20 ± 5.4	61.67 ± 3.33
Monocyte (%)	2.00 ± 0.00	2.00 ± 0.00	1.00 ± 0.00
Eosinophil (%)	2.67 ± 0.33^a^	2.40 ± 0.24^a^	0.67 ± 0.33^b^

Data was analysed using One way ANOVA followed by Tukey's test. The values (in rows) having different letter, in superscript, are significantly different at *p* ≤ 0.05

#### Part II (main study)

The dose 40 μl of 4 × 10^7^ CFU/ml at which less mortality and re-isolation from all the organs were noticed was selected for main study. Another parameter, “the bacterial load”, was also added in the main experiment.

The effect of treatment with plants extract in mice is presented in Table 3. Body weight of the animals at the end of the experiment was significantly less in infection control group/positive control group, however, in the treated groups there was no difference in the final body weight of the animals. In positive control group mortality was 16% (*n* = 1/6) in contrast to 0% in all treated except *A. officinalis* where 50% mortality rate was noted at 5 dpi. Inoculated strain could only be isolated from heart of *A. officinalis* and blood of *Z. jujuba* and *A. officinalis* treated animals.

**Table 3:** Main study: Effects of plant extract treatment on clinical outcome of infected animals at 8 days post infection

Parameters	Groups						
	Positive control	Negative control	Antibiotic	*A. officinalis*	*C. latifolia*	*T. vulgaris*	*Z. jujuba*
Mortality	1/6	0/6	0/6	3/6	0/6	0/6	0/6
Body weight	23.36 ± 0.620	26.83 ± 0.29	25.18 ± 1.05	25.93 ± 0.95	24.65 ± 0.78	25.96 ± 0.85	25.92 ± 1.20
Re-isolation at day 8 post infection							
Throat	6/6	0/6	3/6	3/3	6/6	6/6	6/6
Lung	6/6	0/6	3/6	3/3	6/6	5/6	6/6
Heart	6/6	0/6	0/6	3/3	0/6	0/6	0/6
Joint	6/6	0/6	0/6	0/3	0/6	0/6	0/6
Blood	6/6	0/6	0/6	3/3	0/6	0/6	5/6

#### Gross pathological lesion scoring

The gross lesion score of heart and lung in animals receiving *A. officinalis* was significantly higher (*P* < 0.05) in comparison with animals treated with *C. latifolia*, *T. vulgaris* and *Z. jujuba,* in contrast the lesion score of *C. latifolia*, *T. vulgaris* and *Z. jujuba* treated animals was similar to that of negative control (Table 4).

**Table 4:** Comparison of Gross lesion scores in lungs and heart following treatment of infection with different plant extracts

Groups	Positive control	Antibiotic	Negative control	*A. officinalis*	*C. latifolia*	*T. vulgaris*
Antibiotic	0.034, 0.43					
Negative control	0.034, 0.034	1.00, 0.317				
*A. officinalis*	0.581, 0.197	0.086, 0.068	0.086, 0.034			
*C. latifolia*	0.05, 0.014	1.00, 1.00	1.00, 0.285	0.022, 0.027		
*T. vulgaris*	0.005, 0.005	1.00, 0.157	1.00, 1.00	0.022, 0.005	1.00, 0.138	
*Z. jujuba*	0.005, 0.016	1.00, 0.655	1.00, 0.157	0.022, 0.038	1.00, 0.575	1.00, 0.056

Gross lession score was compared using Kruskle-Wallis test. Comparison among groups was performed using Mann Whitney U test. *P* ≤ 0.05 was considered significant. Data is presented in terms of *P*-values of lungs and hearts respectively

#### Bacterial load estimation

Only *T. vulgaris* treatment resulted in a significant reduction in bacterial load of throat. Likewise, in heart, joint and blood a significant reduction in bacterial load of was observed in *Z. jujuba* and *A. officinalis* treated animals, while from *C. latifolia* and *T. vulgaris* treated animals, the test strain was not recovered (Table 5).

**Table 5:** Effect of treatment with selected medicinal plants on bacterial load of different tissues

Groups	Organs				
	Throat	Lungs	Heart	Joints	Blood
Positive control	4.88 ± 0.20^a^	5.36 ± 0.58^a^	4.52 ± 0.09^a^	3.89 ± 0.23^a^	4.11 ± 0.11^a^
Vancomycin	2.50 ± 1.02^c^	2.50 ± 1.02^c^	0.00 ± 0.00^c^	0.00 ± 0.00^c^	0.00 ± 0.00^c^
*A. officinalis*	4.60 ± 0.13^ab^	4.60 ± 0.13^ab^	3.31 ± 0.43^c^	3.36 ± 0.23^a^	3.94 ± 0.59^b^
*C. latifolia*	4.45 ± 0.20^ab^	4.45 ± 0.20^ab^	0.00 ± 0.00^d^	0.00 ± 0.00^c^	0.00 ± 0.00^c^
*T. vulgaris*	2.93 ± 0.60^bc^	3.83 ± 0.11^abc^	0.00 ± 0.00^d^	0.00 ± 0.00^c^	0.00 ± 0^.^00^c^
*Z. jujuba*	4.44 ± 0.13^ab^	4.44^ab^ ± 0.13	3.77 ± 0.13^b^	1.20 ± 0.76^b^	3.42^b^ ± 0.15^b^

Data was analysed using One way ANOVA followed by Tukey's test. The values (in columns) having no common letter, in superscript, are significantly different at *p* ≤ 0.05. Bacterial load is represented as Mean ± SE of CFU (log_10_)/g of tissue or CFU(log_10_)/ml of the blood

#### Haematological assessment

Infection resulted both in reduction of Hb, RBC, PCV, platelet count and elevation in ESR. While treatment with *C. latifolia*, *T. vulgaris* and *Z. jujuba* indicated therapeutic efficacy by inhibiting changes in aforementioned parameters. *T. vulgaris* treated group showed significant elevation in RBC count which was even more than negative control (Table 6).

**Table 6:** Effect of medicinal plant treatment on hematological parameters of mice at 8th -day post infection

Blood parameter	Groups						
	Positive control	Negative control	Vancomycin	*A. officinalis*	*C. latifolia*	*T. vulgaris*	*Z. jujuba*
ESR mm/h	32.00 ± 1.73^a^	3.67 ± 0.33^e^	6.50 ± 0.33^d^	24.00 ± 0.57^b^	12.33 ± 1.17^c^	13.83 ± 0.47 ^c^	14.50 ± 0.6^c^
Hb g/dL	10.10 ± 1.25^b^	14.67 ± 0.33^a^	14.33 ± 0.33^a^	10.67 ± 0.66^b^	14.67 ± 0.33 ^a^	13.94 ± 0.49 ^a^	14.00 ± 0.025 ^a^
RBC/mm3	6.19 ± 0.80^d^	8.79 ± 0.30^ab^	7.34 ± 0.33^c^	8.00 ± 0.33b^c^	8.17 ± 0.16^bc^	9.33 ± 0.42^a^	8.33 ± 0.33bc
Platelets*1000	129.33 ± 3.48^e^	385 ± 7.63^a^	380.00 ± 22.91^a^	211.67 ± 21.66^d^	385.00 ± 3.41^a^	367.50 ± 8.34^a^	314.17 ± 12.80^b^
PCV %	33.53 ± 4.61^b^	41 ± 0.00^a^	40.00 ± 0.00^a^	40.00 ± 0.00^a^	40.17 ± 0.16a	39.00 ± 0.00^a^	40.33 ± 0.21^a^
TLC *1000	9.0 ± 230.00^a^	5.63 ± 18.62^b^	6.10 ± 20.81^b^	7.13 ± 18.55^b^	6.13 ± 4.94^b^	6.10 ± 7.60^b^	6.25 ± 13.60^b^
Neutrophil (%)	21.00 ± 2.08^a^	10.67 ± 0.42^d^	12.33 ± 1.45^cd^	19.00 ± 3.78^ab^	13.33 ± 1.66^cd^	12.50 ± 0.34^cd^	16.33 ± 1.28^bc^
Lymphocyte (%)	86.33 ± 5.20^a^	71.17 ± 2.55c	72.22 ± 1.20c	81.67 ± 4.41^ab^	82.83 ± 0.91^ab^	83.33 ± 1.66^ab^	80.00 ± 1.26^b^
Monocyte (%)	2.00b ± 0.00	1.33 ± 0.33	1.33 ± 0.33	4.67 ± 0.33	1.00 ± 0.00	2.00 ± 0.00	1.17 ± 0.16
Eosinophil (%)	2.33b ± 0.33^b^	1.2b ± 0.2^b^	1.33 ± 0.33^c^	3.33 ± 0.33^a^	1.00 ± 0.00^c^	1.00 ± 0.00^c^	1.17 ± 0.167^c^

Data was analysed using One way ANOVA followed by Tukey's test. The values (in columns) having no common letter, in superscript, are significantly different at *p* ≤ 0.05. Data are presented in term of Mean ± SE

Similar trend was observed while comparing total and differential leukocyte count of group I-VII. Total leucocyte, neutrophil, lymphocytes and eosinophil counts of mice treated with *C. latifolia*, *T. vulgaris* and *Z. jujuba* were significantly better than positive control group but *A. officinalis* treatment did not yield admirable results. Infection did not alter monocyte count (Table 6).

## Discussion

In vivo studies are crucial for supporting in vitro findings*.* The current study describes in vivo anti-MRSA efficacy of four methanolic plants extracts which are normally used in herbal remedies to control sore throat. Establishing the infection model is the basic requirement of such studies. The dose of bacteria to establish infection depends on the type and virulence of strain, host species, quantity of pathogen and site of inoculation. Therefore, a pilot study was conducted to check pathogenicity and infectious dose of a clinical strain of MRSA in mice.

The experiment revealed that 40 μl dose of 10^7^ CFU/ml was sufficient to induce infection and this infection persisted up to 08 dpi in a murine model. Therefore, this dose was decided for the main experiment. Fu et al. [30] and Cho et al. [31] succeeded in inducing *S. aureus* infection by injection 4 × 10^7^ and 2 × 10^6^ CFU/ml in mice. Arshad et al. [32] established model of collibacillosis by injecting 4.3 log_10_ CFU/ml through intra-peritoneal route.

In main study, ameliorating effects of *C. latifolia*, *A. officinalis*, *T. vulgaris* and *Z. jujuba* were studied. Animals treated with *T. vulgaris* and *Z. jujuba* showed reduction in gross lesion score of both heart and lungs while *C. latifolia* treatment reduced gross lesion in lungs only. Gross lesion develops in tissues due to bacterial virulence. MRSA release toxins which lead to hemolysis in RBC, its adhesion in walls of tissue and resistance to phagocytosis [33]. Reduction in gross lesion score of lungs points towards some role of these extracts in controlling MRSA infection in in vivo system.

*S. aureus* circulates in blood, later on it adheres to components of the extracellular matrix, forming thrombus, or endothelial cells of the host and initiate colonization [34]. It can also interact with vascular endothelial cells [35], extracellular matrix [36] and platelets [37, 38]. Several surface proteins of *S. aureus* have high specificity to matrix and plasma proteins and have been shown to mediate these adhesion processes [39]. *S. aureus* is also known to bind to red blood cells (RBCs) in the presence of plasma proteins such as fibrinogen [40, 41]. Re-isolation and bacterial load from throat, lungs, heart, blood and joint was performed as an indicator of adhesion and persistency of *S. aureus* in tissues. Our data revealed a significant reduction in bacterial load of (a) throat in *T. vulgaris* treated group (b) lungs in antibiotic treated group (c) heart and blood of all treated groups except *Z. jujuba* and *A. officinalis* and (d) joint in *Z. jujuba* and *A. officinalis*. The reduction in bacterial load may be due to bactericidal/bacteriostatic effects of phytochemicals such as alkaloids, flavonoids and phenolic compound present in crude extracts [42]. Our findings are in accordance with Owais et al. [43], who reported a reduction in bacterial load in vital organs of mice after treatment with *Withania somnifera* L. Dunal.

Hematological studies provide a good indication of the progress of bacterial infection as well as treatment. Elevated level of ESR, TLC, neutrophils and lymphocyte count are reliable indicators of infection in the body and they dropped to normal range after effective treatment [44]. *S. aureus* was known to contain hemolysin that can help in targeted killing of host cells. A reduction in Hb, RBC, platelet and PCV was noticed in untreated infected group under study. It may either be due to lysis of RBC during infection or depletion of iron in body indirectly inhibiting erythropoiesis. There are evidences that *S. aureus* is capable of using hemoglobin as a sole iron for growth [45, 46]. Among treated group, except for *A. officinalis*, all plants displayed improvement in haemalogical parametrs. Best adjustment of ESR and lymphocytes was observed after treatment with *C. latifolia*, *T. vulgaris* and *Z. jujube,* in addition, *C. latifolia* and *T. vulgaris* groups had platelet and neutrophil count similar to negative control showing their higher potency in controlling infection. Sur and Ganguly [47] reported a reversal in haematological parameters following treatment with tea plant root extract in Ehrlich ascites carcinoma induced mice. Several studies have reported the antibacterial activity of *A. officinalis* through in vitro procedures [6–8, 45], but its efficiency in in vivo system could not be validated, highlighting the importance of in vivo studies.

## Conclusion

To the best of our knowledge, this is the first report investigating in vivo antimicrobial efficacy of *A. officinalis, Z. jujuba, C. latifolia* and *T. vulgaris*. However, there is no denial to seek for active constituents, their serum-attainable levels, pharmacokinetic properties and diffusion. Current study offered a scientific basis for the medicinal application for *C. latifolia* and *T. vulgaris* extracts against MRSA-associated infections. The antimicrobial activities could be enhanced if their active components are purified and adequate dosage determined for proper administration. This may go a long way in curbing administration of inappropriate concentration; a common practice among many traditional medical practitioners. Secondly, inefficiency of *A. officinalis* in controlling MRSA induced infections emphasizes on employment of in vivo systems for determining therapeutic efficacy of medicinal plants.

## Abbreviations

ANOVA: Analysis of variance
app.: Approximately
CFU: Colony forming units
DLC: Differential leukocyte count
dpi: Days post infection
EDTA: Ethylene diamine tetra acetic acid
ESR: Erythrocytes sedimentation rate
Hb: Hemoglobin
MRSA: Methicillin resistant *Staphylococcus aureus*
PBS: Phosphate buffer saline
PCV: Packed cell volume
RBC: Red blood cell count
SPSS: Statistical program for social sciences
TLC: Total leucocyte count
